# Immunotherapy Effectiveness in Treating Peanut Hypersensitivity: A Systemic Review

**DOI:** 10.7759/cureus.21832

**Published:** 2022-02-02

**Authors:** Rahaf Alghamdi, Rania Alshaier, Aljawharah Alotaibi, Amani Almutairi, Ghadeer Alotaibi, Aisha Faqeeh, Assail Almalki, Hind AbdulMajed

**Affiliations:** 1 Immunology, King Abdulaziz University, Faculty of Medicine, Jeddah, SAU; 2 Immunology, King Abdulaziz University Hospital, Faculty of Medicine, Jeddah, SAU; 3 Tumor Immunology, King Abdulaziz University, Faculty of Medicine, Jeddah, SAU

**Keywords:** non-randomized clinical trial, randomized clinical trial, anaphylaxis, desensitization, immunotherapy, peanut hypersensitivity

## Abstract

Peanut hypersensitivity is one of the top causes of food-related allergic responses and death in high-income countries. As a result, the goal of this study was to see if various forms of immunotherapies can help reduce the severity of peanut hypersensitivity reactions.

From 2019 to 2021, a systematic search of PubMed, Web of Science, Wiley online library, and Science Direct was done. Peanut immunotherapy (PIT) clinical trials were considered. There were 19 trials with a total of 1565 participants. Twelve were on oral immunotherapy (OIT), two on sublingual immunotherapy (SLIT), two on subcutaneous immunotherapy (SCIT), two on epicutaneous immunotherapy (EPIT), and one was a comparison of SLIT and OIT.

Desensitization was achieved by 74.3% of those who received OIT, 11% of those who received SLIT, 61% of those who received SCIT, and 49% of those who received EPIT. The majority of adverse events (AE) were mild to moderate. Those requiring epinephrine, on the other hand, were moderate to severe and were more common in the therapy groups.

This systematic review showed that the current PIT regimens can accomplish desensitization regardless of the route of administration, with an acceptable safety profile.

## Introduction and background

Peanut hypersensitivity is a significant cause of food-related allergic reactions, affecting 2% of children and 1% of adults [1], and was considered the leading cause of food-related lethal anaphylactic reactions in 2014 [1-2]. The Learning Early About Peanut Allergy (LEAP) study found that introducing peanuts to children at a young age reduced the incidence of peanut hypersensitivity and altered the immune response to peanuts in children at high risk of developing the hypersensitivity [3].

The fundamental management of food allergies is to avoid causative foods while waiting for natural tolerance achievement [4]. However, the rate of tolerance achievement for peanuts is low [5-6]. Food hypersensitive patients and their families face several challenges [7], and accidental ingestion is a regular occurrence [8]. Anxiety affects social functioning in people with food allergies, and they have a lower health-related quality of life than people with diabetes [9]. According to that, severe symptoms could be relieved by using some medications such as epinephrine injections and antihistamines [10].

The concept of using immunotherapy for treating peanut hypersensitivity was first introduced by Oppenheimer et al. in 1992. Their study was conducted among 11 participants aged 14-48 years with a history of systemic reaction to peanuts. Results showed a reduction in prick skin reaction (PSR) and clinical symptoms (gastrointestinal, skin, mucosal, respiratory, and systemic) in the peanut immunotherapy (PIT) recipients. However, no change was observed in the placebo recipients [11].

Oral immunotherapy (OIT) is a relatively new therapeutic option for desensitizing youngsters with a range of food allergies. While recent systematic reviews have proven the efficacy of peanut OIT in reaching the immunological endpoint of peanut hypersensitivity desensitization, they have also highlighted major concerns about the treatment's potential dangers [12-13].

One systematic review and meta-analysis was conducted in 2019 by Chu et al. reviewing oral immunotherapy effectiveness and safety in treating peanut hypersensitivity. Despite efficiently achieving desensitization, high-certainty evidence demonstrates that available peanut OIT regimens significantly increase allergic and anaphylactic responses in people with peanut allergies when compared to avoidance or placebo [12].

Systematic reviews have been done to assess the effectiveness of immunotherapy for treating peanut hypersensitivity but to our knowledge, there has not been a comprehensive assessment of the different types of immunotherapies used to treat peanut hypersensitivity.

This systematic review aimed to identify several methods of immunotherapy for treating peanut hypersensitivity, as well as their potential adverse events (AE), and to evaluate its efficacy in hypersensitive participants who were subjected to a specific protocol to improve their symptoms. It also sought to check if there was a difference in peanut hypersensitivity reduction between early and late immunotherapy and if various doses of the same immunotherapy would have different effects.

## Review

Methods

All randomized clinical trials available from January 1, 1989, to June 1, 2021, on different types of peanut immunotherapy as the treatment for peanut hypersensitivity among a wide age range of patients, were included in this systematic review.

Inclusion Criteria

Studies limited to published clinical trials, written in English, including only humans regardless of their gender, age, and nationality, were included.

Exclusion Criteria

Studies involving treatment of atopic diseases other than peanut hypersensitivity or the presence of comorbidities (Severe life-threatening anaphylaxis, including hypotension, cardiovascular diseases, poorly controlled atopic dermatitis, poorly controlled asthma, and eosinophilic gastrointestinal diseases) among the participants were excluded from the analysis.

This review excluded studies where the primary outcome was PIT safety or when the objective was solely about the immunological changes that occur with PIT rather than its efficacy.

Search Process

An extensive search strategy was designed to retrieve all articles published from January 1989 to June 2021 using four electronic bibliographic databases including PubMed, Web of Science, Wiley Online Library, and Science Direct. Articles were reviewed by using the keywords “Immunotherapy”, “peanut hypersensitivity”, “anaphylaxis”, “desensitization”, “randomized clinical trial” and “non-randomized clinical trial”. The titles and abstracts were reviewed as part of the initial screening. In the second screening, studies that met the inclusion criteria and had full-text publications were included. In order to involve a study, two independent reviewers would go over the collected studies from the electronic databases and agree on whether they should be included or excluded. In case of disagreement, a third independent reviewer was consulted to make the final decision.

Data Collection and Analysis

Types of participants: Studies involving patients with peanut hypersensitivity were included. The diagnosis was confirmed through a history of clinical reaction to peanuts, oral food challenge (OFC), or double-blind placebo-controlled food challenge (DBPCFC).

Types of interventions: Studies involving the administration of peanut immunotherapy regardless of the route and dose were included. 

Types of outcomes measured: The primary outcome was measuring the efficacy of immunotherapy by inducing desensitization. Desensitization is defined as the percentage of participants who tolerated the maximum dose of immunotherapy without dose-limiting side effects. This is measured by serological tests, skin-prick test (SPT), and exit OFC. The secondary outcomes were measuring the sustained unresponsiveness, which is defined as the percentage of participants who passed a second OFC after discontinuing PIT for a certain duration determined by the researcher as well as determining the safety of immunotherapy through assessing the frequency and severity of AE by scoring systems, questionnaires, and the use of epinephrine.

Data Extraction

Two independent reviewers extracted the following data from each eligible study: first author name, date of publication, country, number of participants, age range, type of immunotherapy, compared group, maximum tolerated dose, duration, side effect, and evaluated outcomes. Disagreements between reviewers regarding extracted data were resolved through discussion and consensus with a third reviewer.

Assessment of Risk of Bias

The Cochrane risk-of-bias tool was used to assess all included randomized clinical trials independently from six specific domains: selection bias, performance bias, detection bias, reporting bias, attrition bias, and other bias [14].

Statistical Analysis

The Statistical Package for Social Science, version 16 (SPSS Inc., Chicago, IL) was used for data analysis. The analysis was performed to assess the diversity among the included clinical trials. Data were entered separately, and a comparison of the types of immunotherapy, compared groups, total tolerated dose, and duration of the treatment was done.

Result

A total of 3483 studies were found. Four hundred eighty-two studies were published prior to 1989 and were excluded. Initial screening by the title and abstract excluded 2914 studies that were irrelevant to the main objective. Of the remaining 87 studies: 20 did not conform to the primary objective, 15 used non-allergen-based immunotherapy, 12 were duplicated, five included other types of food allergies, five were done on animals, and four were not clinical trials. Seven full texts were not available, of which two were pilot studies, two were inaccessible, one was a protocol, one is ongoing, and one was reporting the first phase of a trial that was then completed and included in this study.

**Figure 1:**
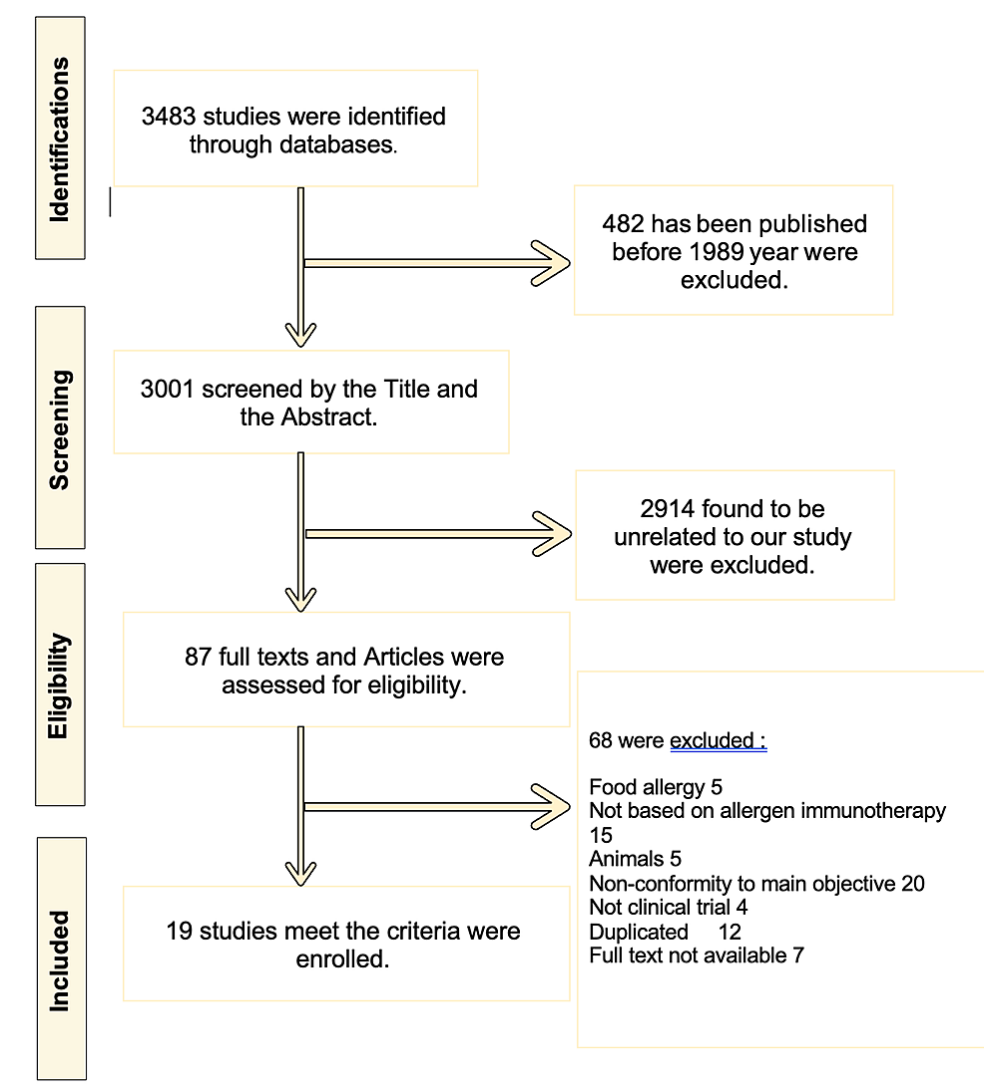
Flowchart of the systematic literature research and selection process

Nineteen studies, as shown in Figure 1, were enrolled in this systematic review, which assessed the efficacy of different routes of receiving PIT among a wide age range of participants [10-11,15-30]. The characteristics of each trial included are summarized in Table 1. Briefly, the trials enrolled a total of 1565 participants (mean number of participants across all studies was 82, with ages ranging from nine months to 56 years) undergoing PIT (12 OIT trials, two SLIT trials, two EPIT trials, two SCIT trials) with the results either being compared to a control group (placebo, avoidance) or not. One study comparing OIT effectiveness to SLIT was included.

**Table 1:** Summary of the included studies in the present systemic review N = Number of participants. SCIT = Subcutaneous Immunotherapy. OIT = Oral Immunotherapy. SLIT = Sublingual Immunotherapy. EPIT = Epicutaneous Immunotherapy. PsIgE = Peanut-Specific Immunoglobulin.

Outcomes	Side effects	Duration	Maximum tolerated dose	Compared group	Type of immunotherapy	Total Participants (Age)	
This study was terminated due to a formulation error that led to the death of one participant. By the time of termination, it was noted that there was a 67% reduction-complete loss of clinical symptoms.	skin reactions, respiratory symptoms, anaphylaxis	4 weeks	500 mg	Placebo (N= 4)	SCIT (N= 4)	14 - 43 years (N= 8)	[11] Oppenheimer J J, et al. 1992, US
Injections of peanut extract increase the tolerance in 25% of participants to oral ingestion of peanuts. Regarding safety, there were high rates of developing systemic reactions and emergent hospital visits.	skin reactions, respiratory symptoms, systemic reactions	12 months	500 mg	Untreated (N= 6)	SCIT (N=6)	18-56 years (N=12)	Nelson HS et al. [30] 1997, US
69% of the participants achieved desensitization. Humeral and cellular changes were suggestive that peanut OIT induces long-term tolerance.	respiratory symptoms, gastrointestinal symptoms, skin reactions.	36 months	1800mg	-	OIT (N= 39)	1-16 years (N=39)	Jones S et al. [22] 2009, UK, US.
OIT was well-tolerated and confirmed protection against at least 10 peanuts, which is more than that is likely to be encountered during accidental ingestion.	systemic reactions, anaphylaxis	28 weeks	800mg	-	OIT (N=4)	9-13 years (N=4)	Clark A et al. [21] 2009, UK
25% of results show a reduction in skin prick test and a significant increase in peanut-specific IgG4 level after using OIT. which will provide protection against accidental exposure.	respiratory symptoms, skin and mucosal reactions, gastrointestinal symptoms	48weeks	4000mg	Placebo (N= 9)	OIT ( N=19)	1-16 years (N=28)	Varshney P et al. [31] 2011, US
All participants who received SLIT achieved desensitization with no significant immunological changes. Oropharyngeal itching was the most common AE reported.	skin reactions, gastrointestinal symptoms, respiratory reaction	18 months	2mg	Placebo (N= 7)	SLIT (N=11)	1-11 years (N=18)	Edwin H. Kim et al. [27] 2011, US
24.2 participants achieved desensitization over a six-month period after starting OIT. safety proﬁle was measured by a disease-speciﬁc questionnaire that showed improvement after intervention and most adverse events were mild.	skin and mucosal reactions, respiratory symptoms, gastrointestinal symptoms	26 weeks	800mg	Avoidance (N=50)	OIT (N=49)	7-16 years (N=99)	Anagnostou K et al. [15] 2014, UK
After peanut OIT 50% recorded sustained unresponsiveness up to 5 years. Safety was assessed by questionnaire and a small portion of participants reported mild side effects.		5 years	4000 mg	-	OIT (N=24)	1-16 years (N=24)	Vickery BP et al. [24] 2014, US
rush OIT protocol was effective with significant changes in immunological responses demonstrated by IgE and IgG4 antibodies ratio to Ara h 2 OIT seems to be relatively safe, and two patients experienced anaphylaxis.	gastrointestinal symptoms, skin reactions, respiratory symptoms	3 years	7000mg	-	OIT (N=16)	9-14 years (N=16)	Nozawa A et al. [20] 2014, JPN
In comparison to SLIT, OIT was more effective in achieving desensitization (14% of the participants). Safety profiles of both immunotherapies have shown mild to moderate adverse events which significantly affected the OIT group more.	gastrointestinal symptoms, systemic reaction	12 months	in SLIT 3.7mg In OIT 2000mg	OIT (N=11)	SLIT (N=10)	7-13 years (N=21)	Narisety S et al. [26] 2015, US
SLIT was found to induce moderate desensitization with 10.8% of participants achieving sustained unresponsiveness. favorable safety profile with mostly mild oropharyngeal symptoms.	mucosal symptoms	3 years	1000mg		SLIT (N=37)	12-40 years (N=37)	Burks W et al. [28] 2015, US
OIT is effective for patients with a peanut allergy. 67% have achieved desensitization to maximum tolerable dose. A major drawback of this study was its lack of random group allocation of participants.	gastrointestinal symptoms, anaphylaxis	39 months	1225 mg	Avoidance (N= 21)	OIT ( N=39)	6-18 years (N=60)	Kukkonen A et al. [16] 2017, FIN
At both doses tested, 81% of the participants achieved desensitization with a significant decrease in PsIgE levels. The safety was evaluated by monitoring the adverse events which showed only a mild to moderate adverse event.	gastrointestinal symptoms, skin reactions, respiratory symptoms	29 months	3000 mg/d for high dose 300mg/d for low dose	Untreated (N= 154)	OIT (N=32)	9-36 months (N=32)	Vickery B et al. [23] 2017, UK and US
In this dose-ranging trial of peanut-allergic patients, the 250 micrograms peanut patch resulted in a significant treatment response. The safety profile is acceptable with the symptoms being mild and local.		12 months	53 received 0.05mg, 56 received 0.1mg, 56 received 0.25mg	Placebo (N= 56)	EPIT (N=165)	6-55 years (N=221)	Sampson H et al. [25] 2017, NA and Eur
EIPT showed moderate response with the highest response among younger children in association with increased IgG4/IgE ratio. The treatment was safe with only mild and local adverse events.	skin reactions	52 weeks	0.25mg	Placebo (N= 25)	EPIT (N=49)	4-25 years (N=74)	Jones S et al. [29] 2017, US
OIT has proved efficacy by increasing the tolerated dose of peanut protein ingested. Adverse events were mostly moderate with a significant decline in severity.	gastrointestinal symptoms, skin reactions, mucosal reactions, respiratory symptoms, anaphylaxis	68 weeks	1000 mg	Placebo (N= 139)	OIT (N=416)	4-55 years (N=555)	Vickery P et al. [17] 2018 NA
68.1% of participants achieved desensitization with a significant decrease in the PsIgE level.	skin symptoms, gastrointestinal symptoms, respiratory symptoms	2 years	795 mg	Historical avoidance (N= 11)	OIT (N=22)	≥5 years (N=22)	Nagakura K et al. [10] 2018, JPN
Discontinuation or reduction of the maximum tolerable dose of OIT seems to increase the probability of regaining clinical reactivity to peanuts.	gastrointestinal symptoms, skin symptoms	104 weeks	4000mg	Placebo (N= 25)	OIT (N=95)	7-55 years (N=120)	Chinthrajah S et al. [18] 2019, CA, US
OIT using AR101 has proved to be effective by the reduction in IgE-IgG4 ratio in the treatment group as well as the improvement in the quality-of-life questionnaire. There was no significant change in peanut-specific IgE between the two groups.	gastrointestinal symptoms, respiratory skin reactions, anaphylaxis	9 months	1000 mg	Placebo (N= 43)	OIT (N=132)	4-17 years (N=175)	Houribane J et al. [19] 2020, Ireland, Fr, Germany, Italy, Spain, Sweden, UK

**Figure 2:**
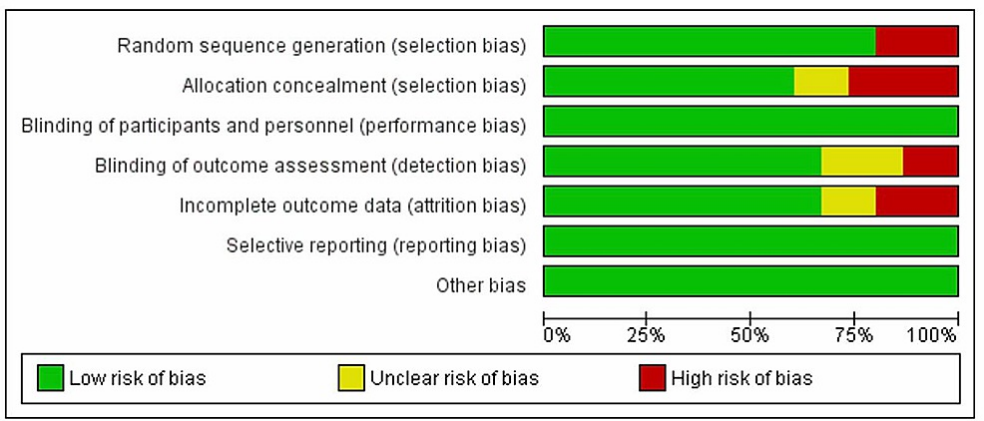
Risk of bias graph The review authors' judgments about each risk of bias item are presented as percentages across all included studies. The highest risk bias was among allocation concealment (65%) while selective reporting got no risk bias; the unclear risk bias was detected in incomplete outcome data, blinding of outcome assessment, and allocation concealment.

**Figure 3:**
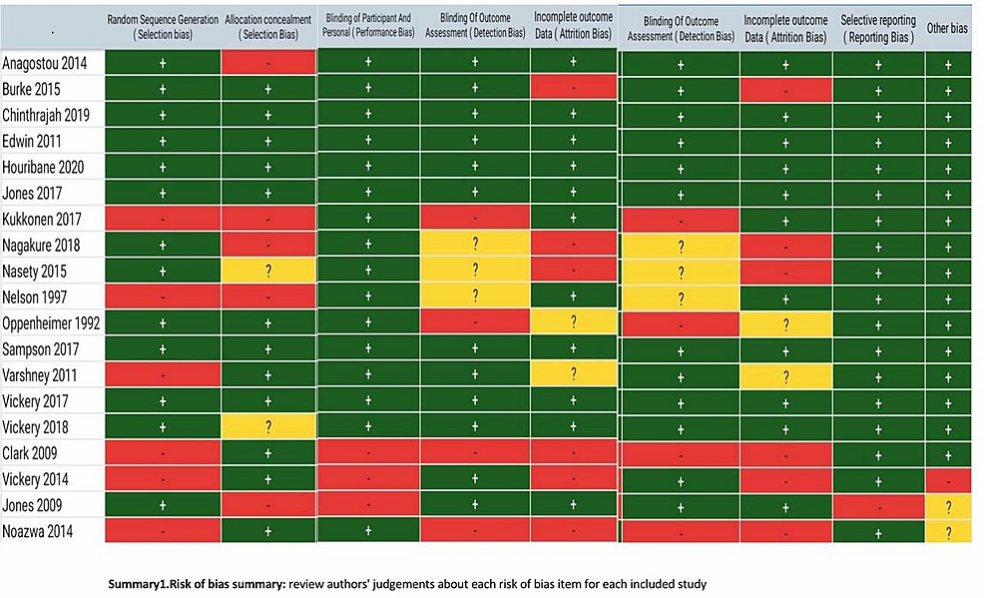
Risk of bias summary The review authors' judgments about each risk of bias item for each included study are depicted. In selective reporting, all studies applied it except Vickery 2014 [29]. We couldn’t determine it in Jones 2009 [24] and Nozawa 2014 [22]. Blinding of participants was applied in all of them except Clark 2009 [23], Vickery 2014 [29], and Jones 2009 [24].

The overall risk of bias of the included studies was low as shown in Figure 2. Domain-specific judgment of each trial is represented in Figure 3.

**Figure 4:**
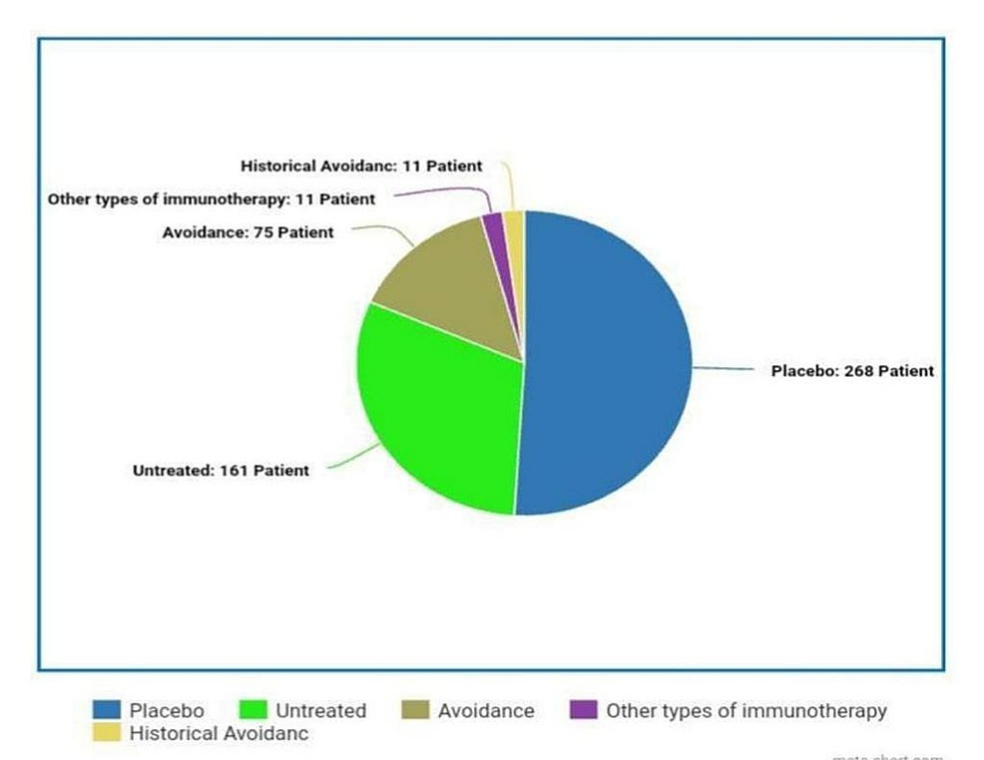
Percentages of the compared groups: placebo, untreated, avoidance, other types of immunotherapy, and historical avoidance Placebo was the most used group (23%), Untreated came second (13.8%) while Avoidance (6.4%), Other Types of Immunotherapy (0.9%), and Historical Avoidance were exactly the same.

**Figure 5:**
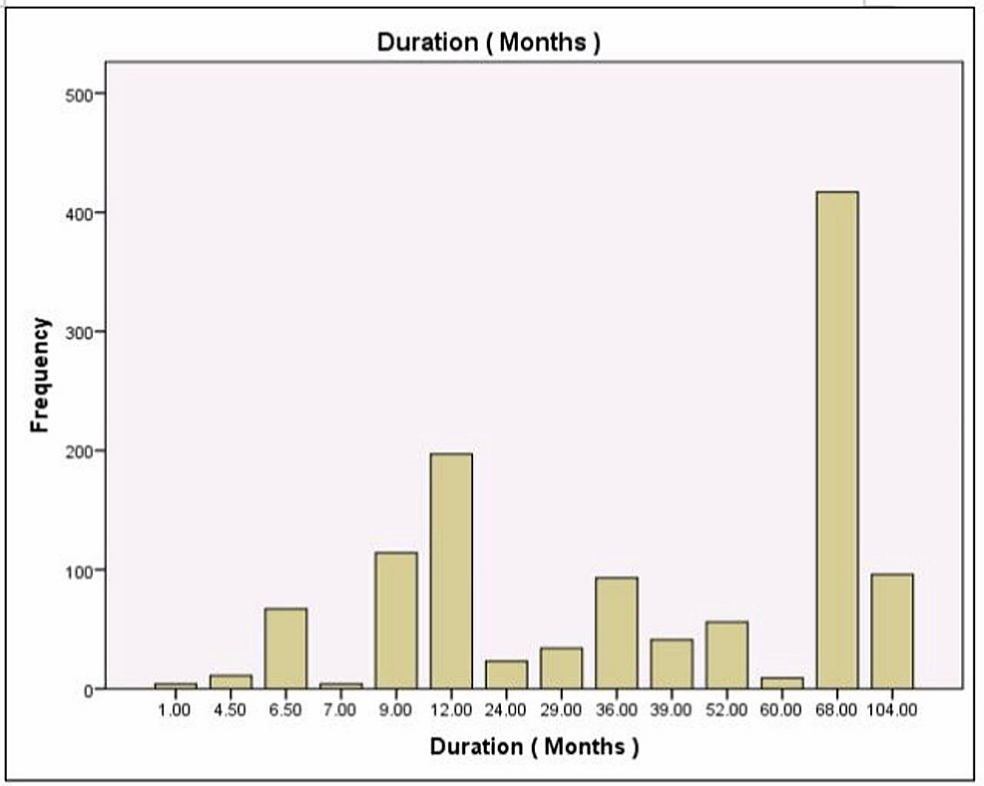
Graph shows the different durations of the therapy The longest duration was 68 months (five years and six months approximately) and the shortest was one month only.

**Figure 6:**
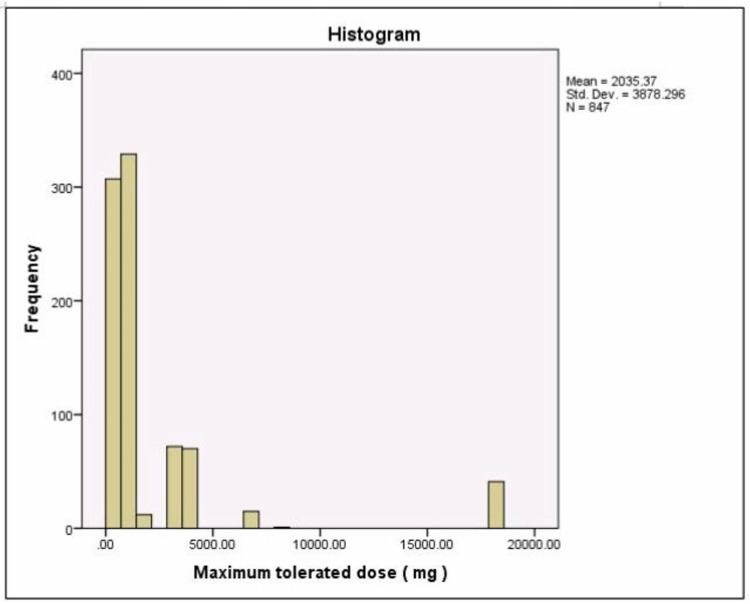
The maximum tolerated dose The mean tolerated dose was 2035.37 mg; most individuals took 100-200 mg. The least dose was 2000 mg and the highest dose was 150,000 mg.

Table 2 presents a statistical analysis of the compared groups while Figure 4 depicts the percentages of the compared groups. Figure 5 shows the different durations of therapy, Table 3 lists the different types of immunotherapy used, Figure 6 shows the maximum tolerated dose.

**Table 2:** Statistical analysis of compared groups Placebo was the most used group (23%); Untreated came second (13.8%); while avoidance (6.4%), Other Types of Immunotherapy (OIT) (0.9%), and Historical Avoidance were exactly the same. OIT = Oral Immunotherapy.

Compared group					
Valid		Frequency	Percent	Valid Percent	Cumulative Percent
	Placebo	268	23.0	51.0	51.0
	Untreated	161	13.8	30.6	81.6
	Avoidance	75	6.4	14.3	95.8
	OIT	11	.9	2.1	97.9
	Historical avoidance	11	.9	2.1	100.0
	Total	526	45.1	100.0	
Missing	System	640	54.9		
Total		1166	100.0		

**Table 3:** Types of immunotherapy given to patients OIT was the most used immunotherapy (75.9%), SCIT was the less used (0.3%), EPIT is the second most common (18.6%), and SLIT was (18.6%). SCIT = Subcutaneous Immunotherapy. OIT = Oral Immunotherapy. SLIT = Sublingual Immunotherapy. EPIT = Epicutaneous Immunotherapy.

Type of immunotherapy					
		Frequency	Percent	Valid Percent	Cumulative Percent
Valid	SCIT	4	.3	.3	.3
	OIT	885	75.9	75.9	76.2
	SLIT	60	5.1	5.1	81.4
	EPIT	217	18.6	18.6	100.0
	Total	1166	100.0	100.0	

Different Types of Immunotherapy Used

**Figure 7:**
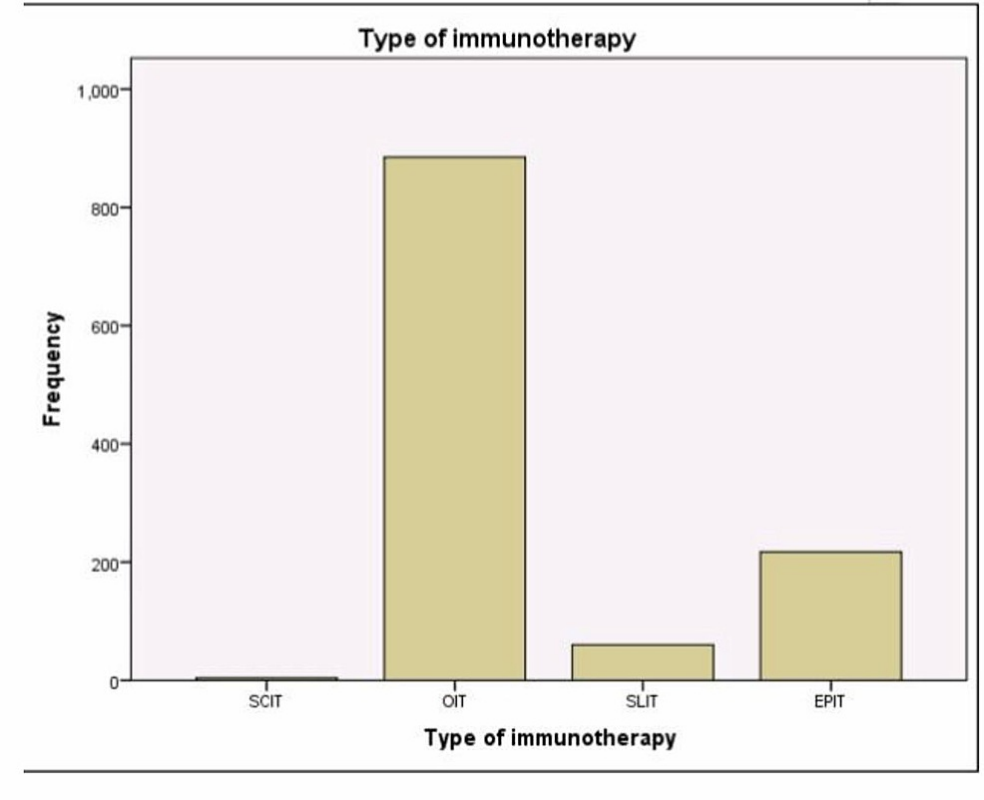
Graph showing the types of therapy OIT was the most used immunotherapy (885), SCIT was the least used (four), EPIT is the second most common (217), and SLIT was in the middle (60). SCIT = Subcutaneous Immunotherapy. OIT = Oral Immunotherapy. SLIT = Sublingual Immunotherapy. EPIT = Epicutaneous Immunotherapy.

 Figure 7 shows a graph of the different types of immunotherapy.

Oral immunotherapy:* *Seven trials reported a higher percentage of participants achieving desensitization and an increase of tolerated peanut threshold [10] among the groups receiving OIT (average of 57%) in comparison to control (average of 0.4%) [15-21,31]. Three trials assessed the clinical improvement in participants receiving OIT and concluded that OIT was both effective and safe in treating peanut hypersensitivity [20] with an average of 85% of participants reaching desensitization [21-22]. One study evaluated the effectiveness of the early introduction of PIT using two different doses of OIT on participants aged from 9-36 months. The trial concluded that early OIT was highly effective in reducing the allergic immune response and achieving safe food reintroduction at both doses evaluated with 81% achieving desensitization [23]. In five trials, OIT successfully induced sustained unresponsiveness in an average of 39% of participants [15,17-18,24,27].

Sublingual immunotherapy: A randomized, double-blind, placebo-controlled experiment revealed that those who received peanut SLIT had a much higher reaction threshold than those who did not [27]. In another study, 40 participants aged 12 to 40 years were assessed for the long-term clinical outcome of SLIT. It was found that 11% of individuals achieved desensitization, with the same number experiencing prolonged unresponsiveness [28]. One trial compared PIT effectiveness between OIT and SLIT. Twenty-one participants aged seven to 13 years were randomized to either active SLIT/placebo-OIT or active OIT/placebo-SLIT in this double-blind trial. Therapy was modified per-protocol after unblinding to provide an extra six months of treatment. Participants who passed the 12 or 18-month challenges were taken off therapy for four weeks before being reassessed. When comparing individuals who received OIT to those who received SLIT, the degree of desensitization was considerably greater. However, only a small percentage of participants remained unresponsive after four weeks of avoidance [26]. Epicutaneous immunotherapy: Two placebo-controlled trials reported that participants who received 250 micrograms had a greater degree of desensitization, with an average of 49%, compared to 19% in the control group. There was no significant difference between the EPIT and control groups at lower doses [25,29]. Subcutaneous immunotherapy: In two trials, long-term PIT was found to be successful in the treatment of peanut hypersensitivity, with an average of 61% of participants in the SCIT group attaining desensitization compared to 50% in the control group [11,30]. *Adverse Events and Epinephrine Use* All of the studies assessed the safety of PIT by evaluating the severity of AE based on direct observation, scoring systems (e.g. Consortium of Food Allergy Research (CoFAR), National Cancer Institute-Common Toxicity Criteria (NCI-CTC)), questionnaires (e.g. Food Allergy Quality of Life Questionnaires (FAQLQ), Food Allergy Independent Measure (FAIM)), or patient diaries. All studies reported mild to moderate AE [24], which most commonly were gastrointestinal AE [16,18-21,23,26-28,31] followed by skin reactions [11,16,22], patch-related local skin reactions [25,29], and respiratory AE [10,30-31]. Three studies found that treatment groups receiving various doses of the same immunotherapy had similar rates of AE in terms of severity and frequency [23,25,29]. One study revealed more AE and early study withdrawal were associated with the OIT group in comparison to the SLIT group [26]. The overall number of trials reporting AE requiring treatment with epinephrine was 13, which ranged from moderate to severe [10-11,15-20,22-23,26,30-31]. No immunotherapy-related death was reported except in one placebo-controlled trial due to a formulation error leading to the termination of the study [11].

Serology and Skin-Prick Test

The majority of the studies measured the serological changes and SPT. Nine studies observed a reduction in peanut-specific immunoglobulin E (PsIgE) [10,18,22-23,25-29], four did not notice a significant change [16-17,19,26] while three reported an increase in its level [11,15,31]. Regarding immunoglobulin G4 (IgG4), 14 studies reported an increase in its level from baseline [10,16-17,19-20,22-29,31]. Two studies observed an increase in the IgG4/IgE ratio [18,29]. Thirteen studies reported a decrease in SPT wheel diameter [11,16-17,19,22-29,31] while one study noticed an increase in it [30].

Discussion

In this systematic review, 3483 clinical trials were screened and only 19 studies, which contained qualitative and quantitative information about assessing the efficacy and safety of PIT in treating peanut hypersensitivity, were selected. No systematic review has done a comprehensive assessment of the several types of immunotherapy used to treat peanut hypersensitivity. Furthermore, very few had compared the different types of PIT.

Across all studies, PIT seems to be effective in achieving desensitization regardless of the type of PIT and the age of participants. However, it was associated with more participants needing to use epinephrine for moderate to severe AE compared to control. AEs were noted to be mainly mild to moderate with the majority reported in OIT trials.

Discussing OIT, Chu et al. reviewed the efficacy and safety of OIT in treating peanut hypersensitivity, which included 12 studies, and found that despite efficiently achieving desensitization, high-certainty evidence demonstrated that available OIT regimens significantly increase allergic and anaphylactic responses in people with peanut hypersensitivity compared to avoidance or placebo [12]. In evaluating the quality of life, two types of questionnaires Food Allergy Quality of Life Questionnaire-Child (FAQLQ-CF) and Food Allergy Quality of Life Questionnaire-Parent (FAQLQ-PF) were used and concluded that OIT may not improve quality of life when compared to avoidance or placebo. In contrast, a study by DunnGlavin et al. used the same types of questionnaires to assess the safety of EPIT [32]. First, it showed that there was a significant improvement in the FAQLQ-PF score over the 24-month period in those who met the primary outcome and those who experienced an improvement in eliciting dose at 12 months. However, there were no significant changes noted in FAQLQ-CF. It is difficult to say with certainty if any of the statistically significant trends in these questionnaires have clinical meaningfulness. Therefore, future studies using objective measures to evaluate the quality of life as a primary outcome should be done.

Although the risk of AE appeared to be consistent across treatment phases, epinephrine use was minimal after patients entered the long-term therapy phase. A systematic review and meta-analysis by Grzeskowiak et al. identified modifiable treatment-protocol-related factors, such as eliminating the rush phase, aiming for a lower target maintenance dose, or using co-treatments in addition to PIT, which could significantly improve the safety and efficacy of treatment regimens and warrant further research [33]. These findings correlate with an included study in this systematic review by Narisety et al., which concluded that a modified treatment regimen appeared to be more effective in achieving desensitization [26]. In contrast, an included study by Chinthrajah et al. found that when compared to avoidance, a lower target maintenance dose did not prevent clinical reactivity [19].

This systematic review showed that current PIT regimens can accomplish desensitization both immunologically and clinically by raising the threshold for the maximum tolerated dose of PIT. Furthermore, it was discovered that AE occurred regardless of the route of administration with an acceptable safety profile. It was also revealed that administering a low dose of the same PIT was just as effective as using a larger dose. This was not the case with EPIT, which showed that only larger dosages were effective. Another observation was that administering PIT to children from a young age was just as safe as giving it later, which encouraged PIT administration earlier. The effectiveness and safety of PIT were the focus of this systematic review. Only clinical trials were included in the evaluation, which matched the study objectives. Relevant information was sought in the PubMed, Science Direct, Web of Science, and Wiley Online Library databases. To check if there were any additional trials, reference lists of relevant publications were searched through. Based on a thematic analysis, the information from the research was synthesized and thematically arranged into topics. Given that this is a review of clinical studies, this technique of analysis was acceptable. These findings highlighted the need for more effective food hypersensitivity treatments with a higher safety profile and clinical studies that focus on patient-relevant outcomes. Several limitations have been discovered at the time of publishing this systematic review; one study was still active. Despite meeting the inclusion criteria, the entire text of two other studies could not be found. Although various studies have been published on PIT, OIT remains the most popular route, with few studies exploring EPIT, SCIT, or SLIT.

## Conclusions

Peanut immunotherapy seems to have effectively induced desensitization in peanut-hypersensitive patients by increasing their tolerance threshold, thus protecting these individuals in case of accidental exposure. However, there were increased incidents of AE among PIT groups compared to avoidance/control. In terms of the safety of PIT, no objective measures of quality of life have been established. Despite the fact that several papers on PIT have been published, OIT remains the most preferred approach compared to other routes. Thus, this systematic review emphasizes the need for more future research focused on the various routes of administration and evaluating their efficacy and safety among different age groups for longer follow-up periods and incorporating the results of the current ongoing study.
